# Fresh Chicken as Main Risk Factor for Campylobacteriosis, Denmark

**DOI:** 10.3201/eid1202.050936

**Published:** 2006-02

**Authors:** Anne Wingstrand, Jakob Neimann, Jørgen Engberg, Eva Møller Nielsen, Peter Gerner-Smidt, Henrik C. Wegener, Kåre Mølbak

**Affiliations:** * Danish Institute for Food and Veterinary Research, Copenhagen, Denmark;; † Statens Serum Institut, Copenhagen, Denmark

**Keywords:** Campylobacter, risk factors, case-control, Denmark, research

## Abstract

We report the findings of a case-control study of risk factors for sporadic cases of human campylobacteriosis in Denmark. In 3 different analytical models, the main domestic risk factor identified was eating fresh, unfrozen chicken. Specifically, 28 of 74 domestically acquired case-patients were exposed to fresh chicken compared with 21 of 114 controls (multivariate matched odds ratio 5.8; 95% confidence interval 2.1–15.9). In contrast, a risk from eating other poultry, including previously frozen chicken, was only indicated from borderline significant 2-factor interactions. The marked increase in consumption of fresh, unfrozen poultry in Denmark during the 1990s likely contributed substantially to the increasing incidence of human campylobacteriosis in this period.

## Abstract 2

Increased consumption of fresh poultry in Denmark has contributed substantially to the increasing incidence of human campylobacteriosis.

*Campylobacter* spp. are the most common cause of acute bacterial gastroenteritis in industrialized countries. Although rarely fatal, *Campylobacter* infections cause considerable illness and loss of productivity and may be associated with severe disabling consequences, including arthritis and demyelinating disease (Guillain-Barré syndrome) (*1*).

Denmark is among a limited number of countries worldwide with comprehensive national laboratory-based surveillance of human campylobacteriosis. Denmark, like several other industrialized countries, has recorded a marked increase in the incidence of human campylobacteriosis. From 1980 to 2001 the incidence quadrupled, reaching 86 cases per 100,000 inhabitants in 2001 (Figure).

**Figure:**
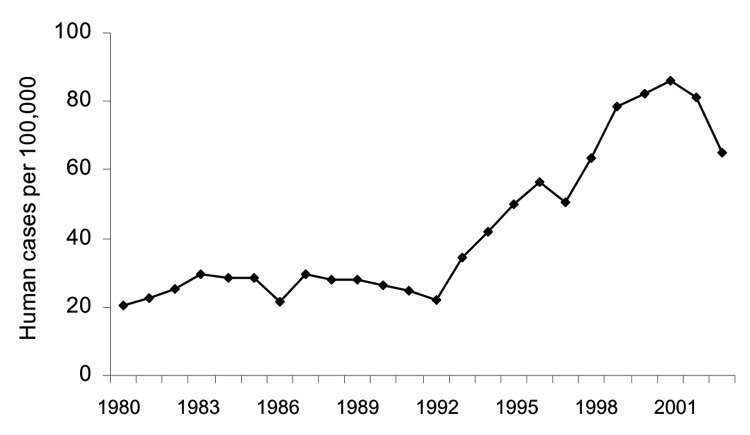
Laboratory-confirmed human campylobacteriosis in Denmark, 1980–2003 (*13*).

Most persons who contract *Campylobacter* infections are not part of recognized outbreaks. Risk factors for sporadic *Campylobacter* infections have been investigated in United States, Canada, Australia, New Zealand, and Europe (including Denmark) within the last 20 years (*2**–**7*). Most studies have identified consumption of poultry and poultry products as risk factors. Other domestic risk factors include drinking untreated water; consuming raw or unpasteurized milk; handling and cooking food, particularly raw meat, in relation to barbecuing; and having contact with food-producing animals and pets.

Although the findings from these studies have provided insight to the epidemiology of *Campylobacter* infections, our understanding is still incomplete. The relative importance of the different sources is not well known, and in many countries, no clear explanation for the increasing incidence of *Campylobacter* infections has been determined. Recent experience from Iceland has pointed to an increased consumption of fresh versus frozen poultry as a potential explanation for the increasing disease incidence (*8*).

The first case-control study of risk factors for human campylobacteriosis in Denmark (*6*) did not distinguish clearly between fresh and previously frozen poultry meat. Several factors, for example, consuming undercooked poultry, but not handling and consuming poultry in general, were risks for human campylobacteriosis. Thawing poultry was found to be protective in this study, which might indirectly indicate that fresh, not frozen, poultry was the main poultry-associated risk factor (*6*).

We report the findings of a second case-control study of risk factors for sporadic human campylobacteriosis in Denmark. In this study, unlike the earlier study, we made a clear distinction in the questionnaire between exposure to fresh, unfrozen meat and exposure to previously frozen meat so we could independently assess the risk of the 2 different categories.

## Materials and Methods

From October 2000 to September 2001, the second case-control study on acute sporadic human campylobacteriosis was conducted in Denmark. Participation in the study was voluntary and required written consent. Three groups were interviewed (computer-aided telephone interviews). The first included campylobacteriosis (CB) patients: persons with laboratory-confirmed campylobacteriosis. The second included healthy controls matched to CB patients by age, sex, and geography (6 controls per CB patient were randomly selected through the Danish Civil Registry system after receipt of a signed consent form from a CB patient). Eligible controls received a questionnaire and a consent form by mail. The time between disease onset for the CB patient and the time of interview of controls was sought diminished (mean 27 days). The third group included non-CB bacterial gastroenteritis patients (non-CB controls): patients whose specimens were culture-positive for other zoonotic bacterial infections (mainly *Salmonella* spp.).

Twice a week, 2–4 CB patients and 3 non-CB patients per CB patient were selected in 6 of 16 Danish counties among patients identified the previous week. An approximate match of non-CB controls to CB patients by onset date was obtained (mean 5 days apart). Children <1 year of age were omitted. Recruitment of patients increased during summer when the incidence was higher. A total of 272 persons with *Campylobacter* infection, 786 non-CB controls, and 2,403 healthy controls were invited for the study. The response rates were 50% for CB cases and non-CB controls and 22% for the healthy controls, respectively.

The questionnaire sought data on the following subjects: symptoms; other diseases; use of medications; use of vitamins; consumption of meat (including type, storing [frozen vs. not frozen], and handling); use of barbecue grill; consumption of rice and pasta, fruit, vegetables, cereals, bread, milk, milk products, spices and herbs, and organic products; cooking; kitchen hygiene; vacation or travel experiences; contact with ill persons; information on drinking water, swimming, household, place of residence, and whether a summer house was used; and respondent's occupation, hobbies, and socioeconomic data. More than 350 original or recoded variables were tested in the analysis.

Three analyses were conducted: A) 107 CB patients versus 178 matched healthy controls (including travel-related cases); B) 74 CB patients (domestically acquired only) versus 114 matched healthy controls; and C) 141 CB patients versus 386 non-CB controls matched to patients only on time of disease onset. Initially, variables in analyses A and B were tested by univariate conditional logistic regression (PROC PHREG, SAS Institute, Cary, NC, USA [9]). Variables with p_(univariate)_<0.30 and other relevant variables were selected for multivariate conditional logistic regression analysis. Variables with p<0.01 were kept in the final models. In analysis B, 2-factor interactions between variables in final model, match variables, and other interactions relevant to the hypotheses were tested; the population attributable risk (PAR) was then estimated (*10*). In analysis C, univariate logistic regression (PROC GENMOD, SAS Institute [9]) was used for screening of effect of variables. Effect modification of covariates (age, sex, geography, and season) was tested in analysis C. As the modifying effect was negligible, the results from analyses without covariates is presented (Table 1).

**Table 1:** Risk factors obtained from 3 analytic approaches for sporadic campylobacteriosis (CB) in Denmark, 2000–2001*

Variables†	Analysis A‡§: 107 CB patients vs. 178 matched controls (multivariate model), OR (95% CI), p	Analysis B§¶: 74 Domestic CB patients vs. 114 matched controls (multivariate model), OR (95% CI), p	Analysis C#: 141 CB patients vs. 386 non-CB controls (univariate analysis), OR (95% CI), p
Travel (14 d)			
Southern Europe	15.81** (2.63–94.9), 0.003	–	0.57 (0.29–1.11), 0.097
Europe, other	0.068** (0.007–0.64), 0.019	–	0.33 (0.11–0.97), 0.044
Outside Europe	16/107 CB, 1/178 controls,** infinite	–	0.93 (0.55–1.55), 0.77
Holiday other than weekend	0.37 (0.13–1.09), 0.072	0.23** (0.083–0.64), 0.005	0.92 (0.62–1.35), 0.67
Chicken bought unfrozen	6.03** (2.17–16.80), 0.0006	5.80** (2.11–15.93), 0.0006	2.91 (1.85–4.59), <0.0001
Turkey††	0.047** (0.004–0.51), 0.012	1.40 (0.59–3.34), 0.45	0.85 (0.54–1.34), 0.47
Turkey bought fresh	61.1** (4.35–857.6), 0.002	1.75 (0.62–4.93), 0.29	1.43 (0.79–2.52), 0.21
Minced beef/veal at barbecue	0.30 (0.065–1.36), 0.12	0.12 (0.018–0.74), 0.022	0.70 (0.28–1.76), 0.45
Pork prepared in large pieces	0.10** (0.028–0.37), 0.0005	0.15** (0.046–0.49), 0.002	0.53 (0.29–0.96), 0.035
Any white bread without bran or grains last month	2.55 (0.92–7.08), 0.072	3.21 (1.15–8.94), 0.026	1.28 (0.73–2.22), 0.39
White bread with bran or grains >5 times/mo	4.54** (1.83–11.23), 0.001	3.10 (1.30–7.35), 0.0105	1.45 (0.97–2.17), 0.074
Apples and pears	0.12** (0.034–0.40), 0.0007	0.21** (0.07–0.61), 0.004	0.83 (0.54–1.27), 0.39
Strawberries‡‡	0.22**(0.069–0.72), 0.012	1.69 (0.60–4.80), 0.32	1.28 (0.75–2.19), 0.37
Raw vegetables daily	0.16**(0.051–0.51), 0.002	0.24**(0.082–0.71), 0.0099	0.93 (0.55–1.56), 0.78
Chives (fresh)	1.94 (0.76–4.95), 0.17	2.78 (1.10–6.99), 0.030	1.19 (0.79–1.77), 0.40
Urban residency	0.17** (0.050–0.58), 0.004	0.25 (0.074–0.85), 0.027	0.76 (0.45–1.31), 0.32
Tap water from summerhouse	4.68 (1.11–19.8), 0.036	3.00 (0.80–11.3), 0.10	0.80 (0.40–1.61), 0.53

*Variables with p<0.05 in the final models from analyses A, B, or both, are shown. †Focus period is the week before onset or filling in questionnaire if nothing else is mentioned. ‡All CB patients (including travelers vs. healthy controls). §Match of patients and controls: age, sex, geography, and as far as possible, to time of disease onset for patient. The risk from factors not included in the final model was achieved by forcing them into the model 1 by 1. ¶Only domestic CB patients vs. healthy controls. #CB patients vs. non-CB bacterial gastroenteritis patients, match to patients only on time of disease onset. **Variables in final models A and B. ††Modifies the risk from turkey bought fresh in model A. ‡‡Modifies the risk from travel to southern Europe in model A.

## Results

Analysis A (full dataset) identified that travel to southern Europe (odds ratio [OR] 15.81) and outside Europe (16/107 patients exposed vs. 1/178 controls) was associated with *Campylobacter* infection, whereas travel to other parts of Europe was more common in controls than in patients (OR 0.068). Other risk factors identified in analysis A are listed in Tables 1 and 2. Analysis B (domestic cases only) identified eating chicken, bought fresh and not frozen in the home, to be the only significant risk factor for campylobacteriosis (OR 5.80). Some exposures, including pork prepared in large pieces (OR 0.15), eating apples or pears (OR 0.21), eating raw vegetables daily (OR 0.24), and days off (besides weekends) in the week before onset (OR 0.23) were more common in controls than patients (p<0.01). Analysis C (CB vs. non-CB patients) found that, among the variables with p<0.05 in analysis A or B, only eating chicken, bought fresh and not frozen in the home, was significantly more associated with *Campylobacter* infections than with other bacterial gastrointestinal infections. Contrary to this finding, travel to central and northern Europe and eating pork prepared in large pieces were less associated with *Campylobacter* infection than with other infections. The domestic PAR from chicken bought unfrozen was 23.8% (95% confidence interval 7.98–52.9).

**Table 2:** Case-control study on sporadic campylobacteriosis (CB) in Denmark, 2000–2001*

Variables	Exposures					
	Analysis A† (including travelers)		Analysis B‡ (only domestic cases)		Analysis C§ (case-case study)	
	107 CB patients, no. (%)	178 matched controls, no. (%)	74 CB patients, no. (%)	114 matched controls, no. (%)	141 CB patients, no. (%)	386 non-CB controls, no. (%)
Travel (14 d)						
No travel	78 (72.8)	156 (87.6)	74 (100)	114 (100)	100 (70.9)	241 (62.4)
Southern Europe	11 (10.2)	6 (3.3)	–	–	4 (2.8)	29 (7.5)
Europe, other	2 (1.8)	15 (8.4)	–	–	12 (8.5)	51 (13.2)
Outside Europe	16 (14.9)	1 (0.5)	–	–	25 (17.7)	65 (16.8)
Holiday other than weekend	49 (45.7)	91 (51.1)	22 (29.7)	52 (45.6)	65 (46.0)	186 (48.2)
Chicken bought unfrozen	36 (33.6)	40 (22.4)	28 (37.8)	21 (18.4)	46 (32.6)	55 (14.2)
Turkey	29 (27.1)	57 (32.0)	23 (31.0)	33 (28.9)	34 (24.1)	105 (27.2)
Turkey bought fresh	28 (26.1)	35 (19.6)	15 (20.2)	15 (13.1)	21 (14.9)	42 (10.9)
Minced beef/veal at barbecue	4 (3.7)	21 (11.7)	3 (4.0)	9 (7.8)	6 (4.3)	23 (6.0)
Pork prepared in large pieces	11 (10.3)	46 (25.8)	8 (10.8)	28 (24.5)	15 (10.6)	71 (18.4)
Any white bread without bran or grains last month	92 (86.0)	142 (79.7)	65 (87.8)	86 (75.4)	122 (86.5)	322 (83.4)
White bread with bran or grains >5 times/mo	75 (70.0)	100 (56.1)	52 (70.2)	62 (54.3)	94 (66.6)	224 (58.0)
Apples and pears	76 (71.0)	148 (83.1)	55 (74.3)	98 (85.9)	98 (69.5)	283 (73.3)
Strawberries	16 (14.9)	43 (24.1)	18 (24.3)	22 (19.2)	23 (16.3)	51 (13.2)
Raw vegetables daily	16 (14.9)	46 (25.8)	10 (13.5)	29 (25.4)	23 (16.3)	67 (17.4)
Chives (fresh)	38 (35.5)	58 (32.5)	30 (40.5)	34 (29.8)	53 (37.6)	130 (33.7)
Urban residence	88 (82.2)	158 (88.7)	61 (82.4)	99 (86.8)	118 (83.7)	336 (87.0)
Tap water from summerhouse	9 (8.4)	12 (6.7)	8 (10.8)	7 (6.1)	11 (7.8)	37 (9.6)

*Exposures for variables with p<0.05 in the final models from analysis A, B, or both. †Analysis A: all CB patients (including travelers) vs. healthy controls matched to patients by age, sex, geography, and as far as possible, to time of disease onset for the patient. ‡Analysis B: domestic CB patients vs. healthy controls matched to patients by age, sex, geography, and as far as possible, to time of disease onset for the patient. §Analysis C: CB patients vs. non-CB bacterial gastroenteritis patients, match to patients only on time of disease onset.

Only in model B (domestic patients) were 2-factor interactions examined. The risk from fresh chicken was significantly increased (p<0.05) in summer (vs. winter) and when preparing whole chicken (vs. cuts). The risk was reduced (p<0.05) by frequently eating fruits, raw vegetables, high-fiber cereals, vitamins (p = 0.050), and acidified milk products (p = 0.070). Eating turkey bought fresh and chicken in general interacted borderline significantly with season (chicken: higher risk in summer [p = 0.078], turkey: higher risk in winter [p = 0.056]). A borderline significant interaction between risk from chicken cuts and barbecuing was found (p = 0.0502). Finally, the apparent protection from eating apples or pears was stronger in the cold season (p = 0.043).

## Discussion and Conclusion

We found that the main domestic risk factor for campylobacteriosis is eating chicken meat that is bought fresh and subsequently not frozen in the home. Eating other poultry meat products and eating previously frozen chicken meat were borderline significant risk factors.

Adding the case-case approach to the risk factor study (CB patients vs. non-CB patients) was expected to highlight risk factors or potentially protective factors, which are specific for campylobacteriosis. Only exposure to unfrozen chicken remained a significant risk factor for campylobacteriosis in the case-case study. The study findings strongly support the contribution of fresh poultry specifically as a source of human campylobacteriosis. In contrast, true common factors for both case groups were expected to be reduced or disappear. Also the apparent effect of factors associated with willingness to participate as a control in the case-control studies was expected to be eliminated. Several significant risk factors from the case-control studies were insignificant or markedly reduced in the case-case study (e.g., apparently protective factors [certain fruits and vegetables] and risk factors [travel, certain types of bread, and fresh turkey]).

The results of the present study are consistent with the hypothesis that a marked increase in the consumption of fresh chicken has been a major driving force behind the increasing incidence of human campylobacteriosis in Denmark during the 1990s. Bacteriologic investigation of fresh and frozen chicken collected at retail outlets in Denmark has shown that the number of viable *Campylobacter* bacteria in fresh samples exceeds that of previously frozen chicken. In a survey of chicken meat in retail stores, 194 (79.8%) of 243 samples of frozen chicken harbored <0.4 thermophilic *Campylobacter* bacteria per gram, whereas 134 (46.4%) of 289 samples of fresh chicken were below this level (*11*). This result is because the freezing process reduces the number of viable *Campylobacter* organisms. In the 1990s, the national consumption of poultry meat increased by ≈40% (1991: 63,900 tons, 1998: 93,200 tons) (*12*). The increase was observed for almost all types of chicken and turkey products but most markedly in fresh cuts. In the same period, the incidence of campylobacteriosis increased by >400%, from 20 to 86 cases per 100,000 inhabitants. The bacteriologic data, which show higher loads of *Campylobacter* in fresh poultry, suggest that the exposures to *Campylobacter* spp. have increased much more than the general increase in poultry consumption and thus explains why the increase in human disease incidence has exceeded the increase in poultry consumption.

The Danish broiler industry, in collaboration with governmental institutions, introduced a voluntary control program in 2002–2003, whereby among other initiatives, flocks of chicken are tested for *Campylobacter* spp. immediately before slaughter (*13*). Positive flocks are, to the extent that doing so is logistically feasible, used to produce frozen products, whereas *Campylobacter*-free flocks are primarily used to produce fresh chicken. In the winter, the prevalence of *Campylobacter*-free flocks is sufficiently elevated to enable a near complete separation, but in the summer, when the flock prevalence is high, *Campylobacter*-positive flocks are also included in the fresh product line to some extent. In 2002, the incidence of human campylobacteriosis dropped 5% from the year before and in 2003 another 19%, possibly as a result of the control program (Figure). Thus, the program appears to have a positive effect, which lends further support to the hypothesis.

In conclusion, the results of this study support the hypothesis that fresh chicken is the main risk factor for domestically acquired campylobacteriosis in Denmark. This risk is significantly increased in the summer, when the incidence of infected broiler flocks peak, and when whole chickens are prepared. Travel to southern Europe and travel outside Europe, respectively, were also significant risk factors. The marked increase in consumption of fresh poultry during the 1990s may explain, at least in part, the increased incidence of human campylobacteriosis in Denmark in this period.
